# LINC01152 upregulates MAML2 expression to modulate the progression of glioblastoma multiforme via Notch signaling pathway

**DOI:** 10.1038/s41419-020-03163-9

**Published:** 2021-01-22

**Authors:** Jianheng Wu, Nannan Wang, Ying Yang, Guangyuan Jiang, Hui Zhan, Fuyong Li

**Affiliations:** 1 Department of Neurosurgery, Gaozhou People’s Hospital, Gaozhou, 525200 Guangdong China; 2 Department of Gastroenterology, Gaozhou People’s Hospital, Gaozhou, 525200 Guangdong China; 3 Electroencephalogram Room, Weifang Yidu Central Hospital, Weifang, 262500 Shandong China; 4 Department of Neurosurgery, Nanxishan Hospital of Guangxi Zhuang Autonomous Region, Guilin, 541000 Guangxi China; 5 Department of Neurosurgery, the People’s Hospital of China Medical University (the People’s Hospital of Liaoning Province), Shenyang, 110016 Liaoning China

**Keywords:** Cancer stem cells, Cell biology, Biomarkers

## Abstract

Glioblastoma multiforme (GBM) brings serious physical and psychological pain to GBM patients, whose survival rate remains not optimistic. Long noncoding RNAs (lncRNAs) have been reported to participate in the progression of many cancers, including GBM. However, the mechanism and function of long intergenic non-protein coding RNA 1152 (LINC01152) in GBM are still unclear. In our study, we aimed to explore the function and mechanism of LINC01152 in GBM. Then qRT-PCR analysis was implemented to search the expression of RNAs in GBM tissues and cells. Functional assays such as EdU assay, colony formation assay, TUNEL assay and flow cytometry analysis were conducted to estimate GBM cell proliferation and apoptosis. RNA pull down assay, luciferase reporter assay, RIP and ChIP assays were implemented to search the binding between molecules. As a result, we discovered that LINC01152 was upregulated in GBM tissues and cells. LINC01152 and mastermind like transcriptional coactivator 2 (MAML2) could both play the oncogenic part in GBM. Moreover, LINC01152 positively regulated MAML2 in GBM by sponging miR-466 and recruiting SRSF1. In turn, RBPJ/MAML2 transcription complex was found to activate the transcription of LINC01152 in GBM cells. In conclusion, LINC01152 could upregulate the expression of MAML2 to promote tumorigenesis in GBM via Notch signaling pathway.

## Introduction

Glioblastoma multiforme (GBM) is the most deadly brain tumor and at least 4 in 100,000 people come down with GBM every year^1^. The prognosis of GBM is seriously dim^2^. GBM not only causes serious physical and psychological harm to the patients, but also imposes an economic burden on the whole society^2^. Even though much improvements have been made in surgery treatment, radiotherapy, and chemotherapy, most of GBM patients can live longer than 1 year after diagnosis^3,4^. Recently, molecular mechanism underlying GBM has been explored and target RNAs have been suggested to be implemented into the therapy of GBM^5^. Nevertheless, the mechanism underlying GBM has not been fully studied^6^. Given the malignancy of GBM, the researches on GBM progression become vital important for us. In this study, we intended to study inner mechanism underlying GBM.

Long noncoding RNAs (lncRNAs) have been defined as RNAs without the ability of coding proteins and also possess the length longer than 200 nucleotides^7^. LncRNA dysregulation has been proved to modulate the development of various human diseases, including cancers^8,9^. Besides, lncRNAs have been verified to modulate the growth, migration and invasion of cancer cells, therefore functioning as tumor promoters or suppressers in cancer^9,10^. For example, lncRNA ZFAS1 has been identified as a regulator of colorectal cancer cell migration and invasion and its overexpression connects to shorter overall survival of colorectal cancer patients^11^. LncRNA ENST457720 can function as an oncogene in non-small cell lung cancer^12^. Also, lncRNA H19 can facilitate cell growth by modulating the expression of E2F-1 in pancreatic ductal adenocarcinoma^13^. Meanwhile, long intergenic non-protein coding RNA 1152 (LINC01152) has been found to be highly upregulated and activate tumor formation in hepatocellular carcinoma^14^. Also, we found LINC01152 was highly expressed in GBM and brain lower grade glioma (LGG) according to GEPIA database. Nevertheless, the function of LINC01152 is still unclear in GBM, thence we further studied the function of LINC01152 in GBM.

Protein-coding genes have been regarded as regulators of diverse cancers by affecting cellular biological behaviors^15^. For instance, FABP4 can hamper cell growth and invasion in hepatocellular carcinoma^15^. FOXQ1 upregulated in esophageal cancer can facilitate cell proliferation, migration, and invasion in esophageal cancer by reducing the expression of CDH1^16^. Mastermind like transcriptional coactivator 2 (MAML2) is involved into the progression of many cancers, such as mucoepidermoid carcinoma and breast cancer^17,18^. Also, GEPIA database detected that MAML2 was highly upregulated in GBM. However, the role of MAML2 in GBM is still not clear.

In present study, the role of MAML2 and LINC01152 in GBM was carefully studied. Meanwhile, considering the competing endogenous RNA (ceRNA) network has been well-proposed, we aimed to carefully scrutinize the connection of MAML2 with LINC01152 in GBM.

## Materials and methods

### Tissue collection

Thirty-eight GBM tissue samples were collected from GBM patients and 38 normal brain tissue samples extracted from patients without GBM between April 2014 and July 2019, with the approval from the Ethics Committee of Gaozhou People’s Hospital. None of these patients received chemotherapy or radiotherapy before surgery, and the written informed consents were all signed by the participants. Samples were snap-frozen after the operation in liquid nitrogen and the preserved at −80 °C for RNA extraction.

### Cell lines

The human LGG cell lines (LN-215 and U138) and normal human astrocytes (NHA), as well as GBM cell lines (M059K, LN-229, T98G, and U343) and normal glial cell line (HEB), were all purchased for our research. In detail, U138, M059K, LN-229, and T98G cells were available from ATCC Company (Rockville, Maryland), LN-215 and U343 cells were purchased from Shun Ran Biotechnology Co., Ltd (Shanghai, China), while NHA and HEB cells were obtained from Qin Cheng Biotechnology Co., Ltd (Shanghai, China). As for the culture medium, Dulbecco’s Modified Eagle’s Medium (DMEM) was applied for incubating LN-215, NHA, LN-229, HEB, and U343 cells, Eagle’s Minimum Essential Medium was utilized to cultivate U138 and T98G cells, and M059K cells were cultivated in DMEM and Ham’s F12 medium. All the culture media were supplemented with 10% FBS and 1% mixture of Pen/Strep (Invitrogen, Carlsbad, CA), and all cells were grown under the condition of 5% CO_2_ and 37 °C.

### Quantitative real-time polymerase chain reaction (qRT-PCR)

The cultured cells were treated with Trizol reagent (Invitrogen) for total RNA extraction, and then cDNA synthesis was achieved using PrimeScript™ RT reagent kit in the light of the manufacturer’s instruction (Takara, Shiga, Japan). Gene expression was quantified by qRT-PCR with SYBR Premix Ex Taq II (Takara), and then calculated by the 2^−ΔΔCt^ method and standardized to U6 or GAPDH.

### Plasmid transfection

The shRNAs (GenePharma, Shanghai, China) specific to LINC01152, MAML2, SRSF1, ADAR, RBPJ, and the NC-shRNAs were transfected into T98G and U343 cells as per protocol of Lipofectamine 3000 (Invitrogen). Besides, miR-466 mimics/inhibitor and NC mimics/inhibitor, together with pcDNA3.1-LINC01152, pcDNA3.1-MAML2, pcDNA3.1-RBPJ and pcDNA3.1-NC were procured from RiboBio (Guangzhou, China). After 48 h of transfection, cells were all reaped.

### EdU staining

1 × 10^4^ cell samples were plated to each well of 96-well plates, with the addition of EdU medium for 2 h of cultivation at 37 °C. Samples were fixed by 4% paraformaldehyde, and then processed with EdU staining reagent from Ribobio. DAPI dye was employed to counter-stain the nuclei for five minutes at room temperature, and samples were then observed with a fluorescent microscope as instructed (Olympus, Tokyo, Japan). Experiments were performed in triplicate.

### Colony formation assay

Cell samples were subjected to a 14-day culture in 6-well plates, with 500 cells per well. Following fixing by 4% paraformaldehyde, cell samples were stained by 0.1% crystal violet for 30 min, and then counted manually. Experiments were performed in triplicate.

### TUNEL staining

Apoptotic cells were examined by use of In Situ Cell Apoptosis Detection Kit as guided (Roche, Basel, Switzerland). The fixed cells on coverslips were rinsed in PBS for 15 min, followed by double-staining with TUNEL staining reagent and DAPI dye. Fluorescence pictures were taken under a fluorescent microscope. Experiments were performed in triplicate.

### Flow cytometry

Annexin V-FITC/PI Apoptosis kit was procured from BD Biosciences (San Jose, CA) and used as per user manual. 1×10^6^ cells were collected and fixed on ice for 1 hour. Following double-staining in Binding Buffer for 15 min in dark, cells were analyzed by FACS Calibur flow cytometer (BD Biosciences). Experiments were performed in triplicate.

### Animal study

Six-week-old male BALB/C nude mice were available from Beijing Vital River Laboratory Animal Technology Co. Ltd. (Beijing, China) and used for animal study, with the approval from the Animal Research Ethics Committee of the Medical College of Guilin. 1 × 10^6^ cell samples in PBS were injected subcutaneously to nude mice, and tumor volume was recorded every fourth day. The cells were previously transfected with sh-NC, sh-LINC01152#1, sh-LINC01152#1 + antagomir-466, or sh-LINC01152#1 + pcDNA3.1/MAML2. 28-day after injection, mice were killed by cervical decapitation, and then tumors were excised and weighed for analysis.

### Western blot

After lysing cells in RIPA lysis buffer, total protein samples were acquired, followed by protein separation on 10% SDS-PAGE (Millipore, Bedford, MA, USA) and shifting to PVDF membranes (Millipore). After sealing in 5% nonfat milk, membranes were probed all night at 4 °C with primary antibodies against the internal control GAPDH (ab8245, Abcam) or Tubulin (ab7291, Abcam), as well as MAML2 (ab245612, Abcam), p21 (ab218311, Abcam), HES-1 (ab71559, Abcam), SRSF1 (ab38017, Abcam), ADAR (ab88574, Abcam) and RBPJ (ab25949, Abcam). Following washing in TBST, the HRP-tagged secondary antibody was added for 2 h at room temperature. Finally, ECL luminous liquid (Pierce, Rockford, IL) was added for detection. Experiments were performed in triplicate.

### Luciferase reporter assay

LINC01152 or MAML2 fragments covering miR-466 binding sites (wild-type and mutant) were inserted into pmirGLO luciferase vector (Promega, Madison, WI), and the indicated recombinant plasmid was then co-transfected into T98G and U343 cells with miR-466 mimics or NC mimics for 48 h. Besides, these cells were also co-transfected with pGL3 luciferase vector (Promega) containing wild-type or mutant LINC01152 promoter and indicated plasmids for gene transcription analysis. Samples were subjected to Luciferase Reporter Assay System (Promega) for detection of the luciferase intensity. Experiments were performed in triplicate.

### Subcellular fraction

After washing in precooled PBS, 1 × 10^6^ cells were centrifuged in cell fractionation buffer for acquiring cell cytoplasm. Then, cell disruption buffer was added to collect cell nuclei. LINC01152 content was separately examined via qRT-PCR in both cytoplasmic and nuclear fractions. Experiments were performed in triplicate.

### FISH

The fixed cell samples were digested, dehydrated and air-dried, followed by culturing with LINC01152-specific FISH probe (Ribobio) in hybridization buffer. After adding DAPI dye, samples were monitored under a fluorescent microscope. Experiments were performed in triplicate.

### RNA pull down assay

RNA pull down assay in T98G and U343 cells were accomplished by use of Pierce Magnetic RNA-Protein Pull-Down Kit in line with the instruction (Thermo Fisher Scientific, Waltham, MA). Cell extracts were mixed with biotin-tagged probes for LINC01152 or miR-466 and relative control probes. Then, the cell lysates were incubated with Dynabeads ™ M-270 Streptavidin Magnetic Beads (Invitrogen) for pulling down the biotin-labeled RNAs. The magnetic beads were then washed, and the obtained RNAs or proteins were analyzed by qRT-PCR or western blot, respectively. Experiments were performed in triplicate.

### RNA immunoprecipitation (RIP)

After incubation in RIP lysis buffer, cell lysates were conjugated with SRSF1 antibody or human Ago2 antibody in magnetic beads. IgG antibody (Millipore, Billerica, MA) served as the negative control. All precipitated RNAs were assayed by qRT-PCR. Experiments were performed in triplicate.

### Co-immunoprecipitation (CoIP)

After culturing in IP lysis buffer, cell lysates were used to incubate with anti-RBPJ, anti-NICD, anti-MAML2, and anti-IgG (the negative control) antibodies overnight at 4 °C in constant speed. Following elution via magnetic beads, protein samples were examined by western blot. Experiments were performed in triplicate.

### Chromatin immunoprecipitation (ChIP)

The processed cells were first fixed for 15 min cross-link, and then treated by ultrasonic to shear DNA. RBPJ, MAML2, and IgG antibodies, as well as magnetic beads were separately added into samples. Immunoprecipitated DNA was quantified by qRT-PCR. Experiments were performed in triplicate.

### Statistical analyses

All experiments in this study were bio-repeated for three times, data were processed by SPSS version 19.0 software (IBM Corp., Armonk, NY) and exhibited as mean ± standard deviation (SD). The differences of statistical analyses, determined in form of *t*‐test or one‐way ANOVA, was considered significant when *p*-values below 0.05.

## Results

### LINC01152 was upregulated and played an oncogene role in GBM

**Fig. 1:**
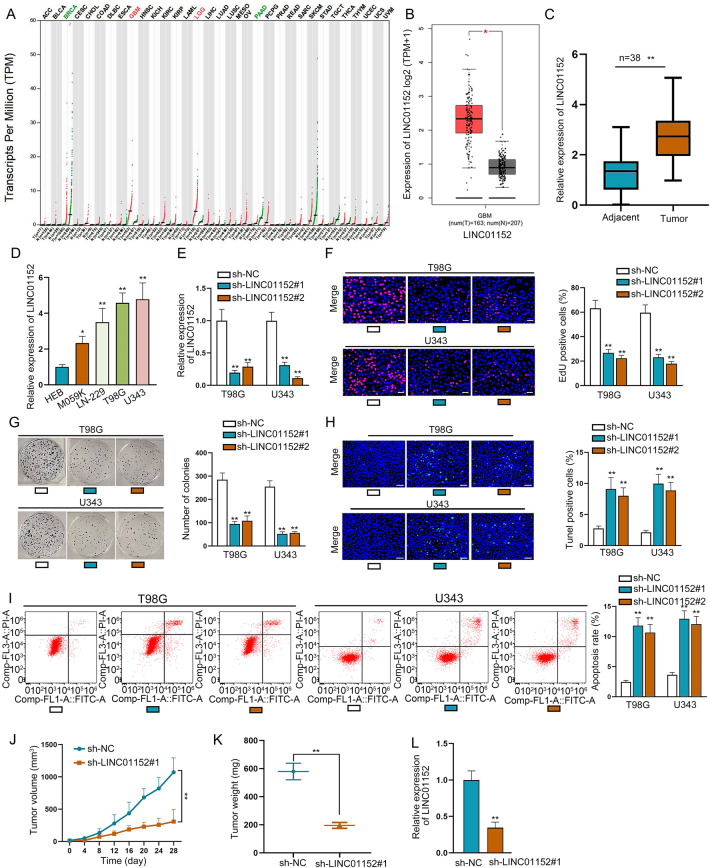
LINC01152 was upregulated and played as an oncogene in GBM. **A** GEPIA (http://gepia.cancer-pku.cn/) database was implemented to search LINC01152 expression pattern in different cancer types including GBM. **B** GEPIA data of LINC01152 level in 163 GBM tissues and 207 normal brain samples. **C** Expression of LINC01152 in 38 pairs of GBM tissues was assessed via qRT-PCR. **D** qRT-PCR was conducted to determine the expression of LINC01152 in normal human glial cell line (HEB) and GBM cell lines (M059K, LN-229, T98G and U343). **E** Inhibition efficiency of LINC01152 in T98G and U343 cells was examined via qRT-PCR. **F**, **G** EdU (scale bar = 100 μm) assay and colony formation assay assessed the proliferation of T98G and U343 cells when LINC01152 was silenced. **H**, **I** TUNEL (scale bar = 120 μm) assay and flow cytometry analysis were implemented to test the apoptosis of T98G and U343 cells when LINC01152 was silenced. **J**, **K** The volume and weight of tumors obtained from in vivo assays. **L** LINC01152 expression was tested in in vivo tumors from two different groups. *P < 0.05, **P < 0.01.

LINC01152 has studied as an oncogene in hepatocellular carcinoma^14^. Meanwhile, LINC01152 expression was identified to be highly expressed in GBM and brain lower grade glioma (LGG) through GEPIA (http://gepia.cancer-pku.cn/) database (Fig. 1A) and LINC01152 was also further unveiled to be upregulated in GBM and LGG tissues (Fig. 1B and Fig. S1A) according to GEPIA database. Meanwhile, we searched the expression of LINC01152 in LGG cells via qRT-PCR analysis, and results indicated that LINC01152 was not significantly highly expressed in LGG cells (LN-215 and U138) compared with the normal NHA cells (Fig. S1B). In our study, we detected the expression of LINC01152 in GBM tissues (*n* = 38) and GBM cell lines (M059K, LN-229, T98G, and U343) via qRT-PCR. It manifested that LINC01152 was upregulated in GBM tissues compared to paired controls and also in GBM cells relative to the normal glial cell line (HEB) (Fig. 1C, D). Thence, we focused on the function of LINC01152 in modulating GBM progression. Then, shRNAs covering interference sequence of LINC01152 were transfected into T98G and U343 cells, and qRT-PCR verified the great silence of LINC01152 in such cells (Fig. 1E). Notably, the results of EdU assay and colony formation assay delineated that the rate of EdU positive cells and the number of colonies were both reduced by silenced LINC01152 (Fig. 1F, G). Also, TUNEL assay and flow cytometry analysis found that the percent of TUNEL positive cells and the apoptosis rate were both increased with LINC01152 inhibition (Fig. 1H, I). Meanwhile, subcutaneous tumor formation assay was also implemented to search the function of LINC01152 inhibition in vivo. Results showed that tumor growth rate and weight were both reduced when the tumors were originated from cells with silenced LINC01152 (Fig. 1J, K). Besides, LINC01152 expression was verified to be lowered in tumors from LINC01152-inhibited GBM cells (Fig. 1L). In brief, results indicated that LINC01152 was upregulated and played an oncogenic role in GBM.

### LINC01152 could promote the progression of GBM by upregulating MAML2

**Fig. 2:**
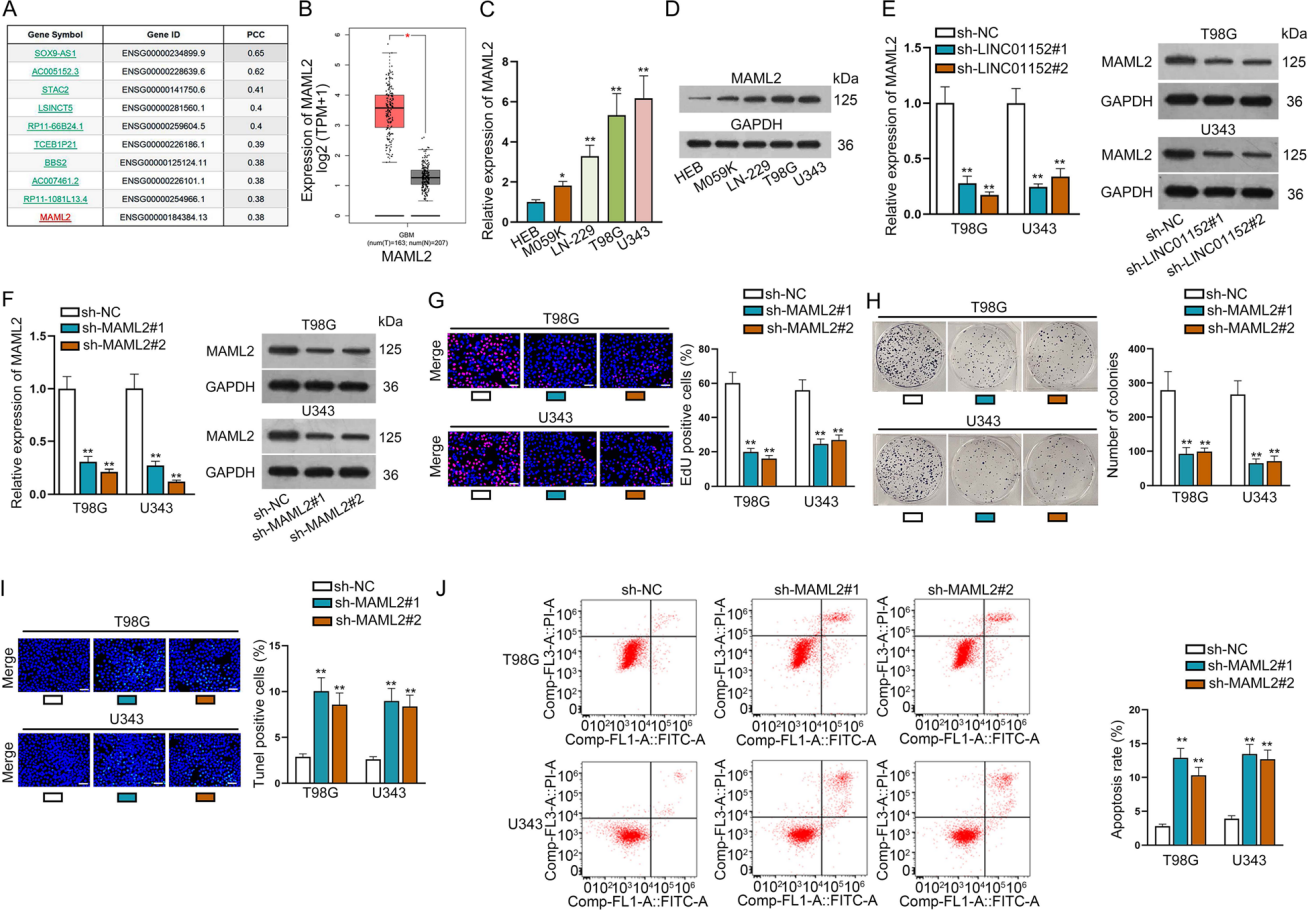
LINC01152 could promote the progression of GBM by upregulating MAML2. **A** LINC01152 related genes were identified via GEPIA database. **B** Data of MAML2 expression in GBM was acquired from GEPIA database. **C**, **D** The mRNA and protein levels of MAML2 in normal human glial cell line (HEB) and GBM cell lines (M059K, LN-229, T98G and U343) were tested via qRT-PCR and western blot, respectively. **E** qRT-PCR and western blot identified the expression of MAML2 when LINC01152 was silenced in T98G and U343 cells. **F** Silence efficiency of MAML2 was verified via qRT-PCR and western blot in T98G and U343 cells. **G**, **H** EdU (scale bar = 100 μm) assay and colony formation assay assessed the proliferation of T98G and U343 cells when MAML2 was silenced. **I**, **J** TUNEL (scale bar = 120 μm) assay and flow cytometry analysis were implemented to test the apoptosis of T98G and U343 cells when MAML2 was silenced. *P < 0.05, **P < 0.01.

To explore the regulatory mechanism of LINC01152 in promoting GBM, we searched LINC01152 related genes via GEPIA database and the top ten were presented in Fig. 2A. In the meantime, the expression of three protein-coding genes among them, including STAC2, BBS2, and MAML2, were specifically searched in GEPIA database. Interestingly, we found that only MAML2 was highly expressed in GBM samples (Fig. 2b and Fig. S1C). Then, we detected the expression of MAML2 in normal GBM tissues and cell s. It was suggested that more MAML2 expressed in GBM tissues compared to adjacent non-tumor ones (Fig. S1D). In addition, we also discovered that MAML2 exhibited high expression in GBM cells relative to HEB cells (Fig. 2C, D). Importantly, we proved that the mRNA and protein levels of MAML2 were both declined when LINC01152 was silenced in T98G and U343 cells (Fig. 2E). Furthermore, we investigated the function of MAML2 in GBM cells after verifying the indeed silence efficiency of MAML2 via qRT-PCR and western blot analyses (Fig. 2F). Cell growth of T98G and U343 cells was found to be inhibited in response to MAML2 depletion (Fig. 2G, H). Also, the apoptosis of these two GBM cells was stimulated by MAML2 depletion (Fig. 2I, J). In conclusion, LINC01152 could promote the progression of GBM by upregulating MAML2.

### LINC01152/miR-466/MAML2 axis was found in GBM cells

**Fig. 3:**
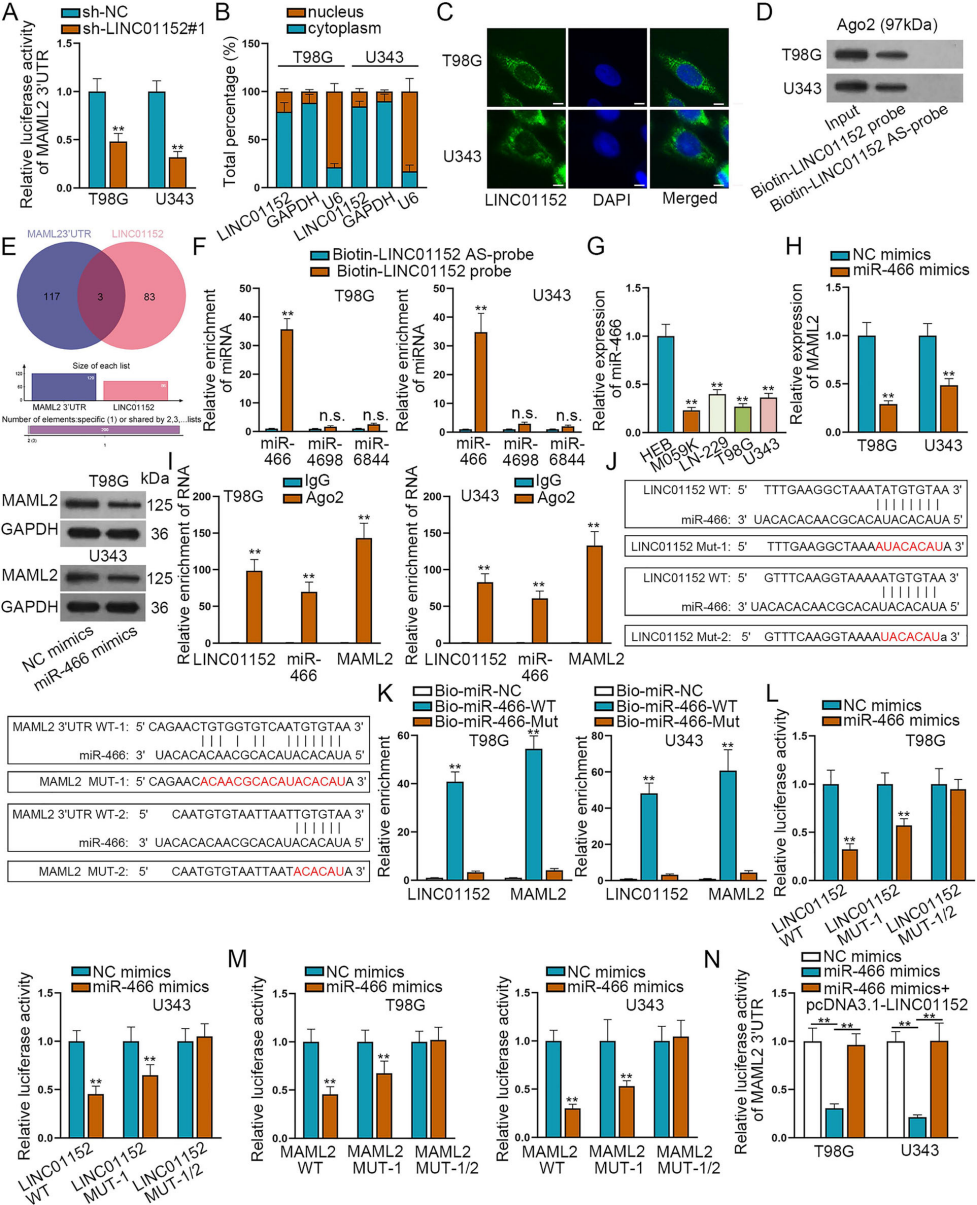
LINC01152/miR-466/MAML2 axis was found in GBM cells. **A** Luciferase reporter assay assessed the luciferase activity of MAML2 3’UTR in T98G and U343 cells when LINC01152 was silenced. **B**, **C** Subcellular fraction assay and FISH assay (scale bar = 20 μm) searched the location of LINC01152 in T98G and U343 cells. **D** RNA-protein pull down assay searched the ceRNA potential of LINC01152 in T98G and U343 cells. **E** MiRDB (http://mirdb.org/) database was conducted to search possible miRNAs who could bind to MAML2 3’UTR and LINC01152. **F** RNA pull down assay searched the binding of miR-466, miR-4698, and miR-6844 to LINC01152 in T98G and U343 cells. **G** Expression of miR-466 was examined in normal human glial cell line and GBM cell lines via qRT-PCR. **H** Expression of MAML2 was tested via qRT-PCR and western blot when miR-466 was overexpressed in T98G and U343 cells. **I** RIP assay searched the axis of LINC01152/miR-466/MAML2 in T98G and U343 cells. **J** The binding sites of miR-466 in LINC01152 and MAML2 3’UTR were presented according to ENCORI (http://starbase.sysu.edu.cn/index.php) database. **K**–**M** RNA pull down assay and luciferase reporter assay detected the existence of LINC01152/miR-466/MAML2 axis in GBM cells. **N** Luciferase activity of MAML2 3’UTR was assessed via luciferase reporter assay in indicated T98G and U343 cells. ^**^P < 0.01, n.s.: no significance.

In order to search the in-depth mechanism whereby LINC01152 affected MAML2 expression in GBM, we then conducted luciferase reporter assay. Results indicated that the absence of LINC01152 had no obvious influence on the luciferase activity of MAML2 promoter (Fig. S1E), but markedly reduced the luciferase activity of MAML2 3’UTR (Fig. 3A). It proved that LINC01152 modulated MAML2 at post-transcriptional level. Thence, we wanted to know the location of LINC01152 in GBM cells. The outcomes of subcellular fraction assay and FISH assay unmasked that LINC01152 located in both the nucleus and cytoplasm, but mainly in the cytoplasm of T98G and U343 cells (Fig. 3B, C). Meanwhile, RNA-protein pull down assay found that Ago2, the core protein of RNA-induced silencing complexes (RISCs), could be abundantly detected in the products pulled down by LINC01152 biotin-probe (Fig. 3D), which proved the ceRNA potential of LINC01152 in GBM. Thence, miRDB (http://mirdb.org/) database was implemented to search possible miRNAs who could bind to MAML2 3’UTR and LINC01152 (Fig. 3E). In this regard, miR-466, miR-4698 and miR-6844 were found out. Then, RNA pull down assay found that only miR-466 was significantly enriched in the pull-down compounds of Biotin-LINC01152 probe (Fig. 3F). Meanwhile, we uncovered that miR-466 presented low expression trend in GBM cells (Fig. 3G), so did in GBM specimens (Fig. S1F). Thereafter, we transfected miR-466 mimics into T98G and U343 cells and testified the overexpression of miR-466 in such cells (Fig. S1G). As anticipated, the expression of both MAML2 mRNA and protein was reduced by overexpressed miR-466 (Fig. 3H). Further, RIP assay results identified that LINC01125, miR-466 and MAML2 were all enriched in RISCs (Fig. 3I). Thence, we presented the binding sites of miR-466 in LINC01152 and MAML2 3’UTR in Fig. 3J, according to the prediction of ENCORI (http://starbase.sysu.edu.cn/index.php) database. Moreover, the outcomes of RNA pull down assay evidenced that both LINC01152 and MAML2 were enriched by Bio-miR-466-WT (Fig. 3K). Meanwhile, it was showed that the luciferase activities ofLINC01152-WT, LINC01152-MUT1, MAML2-WT and MAML2-MUT1 were all decreased by miR-466 overexpression, whereas that of LINC01152-MUT-1/2 or MAML2-MUT-1/2 was not influenced (Fig. 3L, M). Above results proved the direct binding of miR-466 to LINC01152 or MAML2 3’UTR at the predicted sites. To further validate that LINC01152 was a ceRNA of MAML2 in GBM, we then overexpressed LINC01152 in T98G and U343 cells (Fig. S1H). As expected, the decreased luciferase activity of MAML2 3’UTR resulted from miR-466 overexpression was regained in the context of LINC01152 upregulation (Fig. 3N). In a word, LINC01152 served as a ceRNA of MAML2 in GBM through absorbing miR-466.

### LINC01152 partially depended on miR-466 to modulate the behaviors of GBM cells

**Fig. 4:**
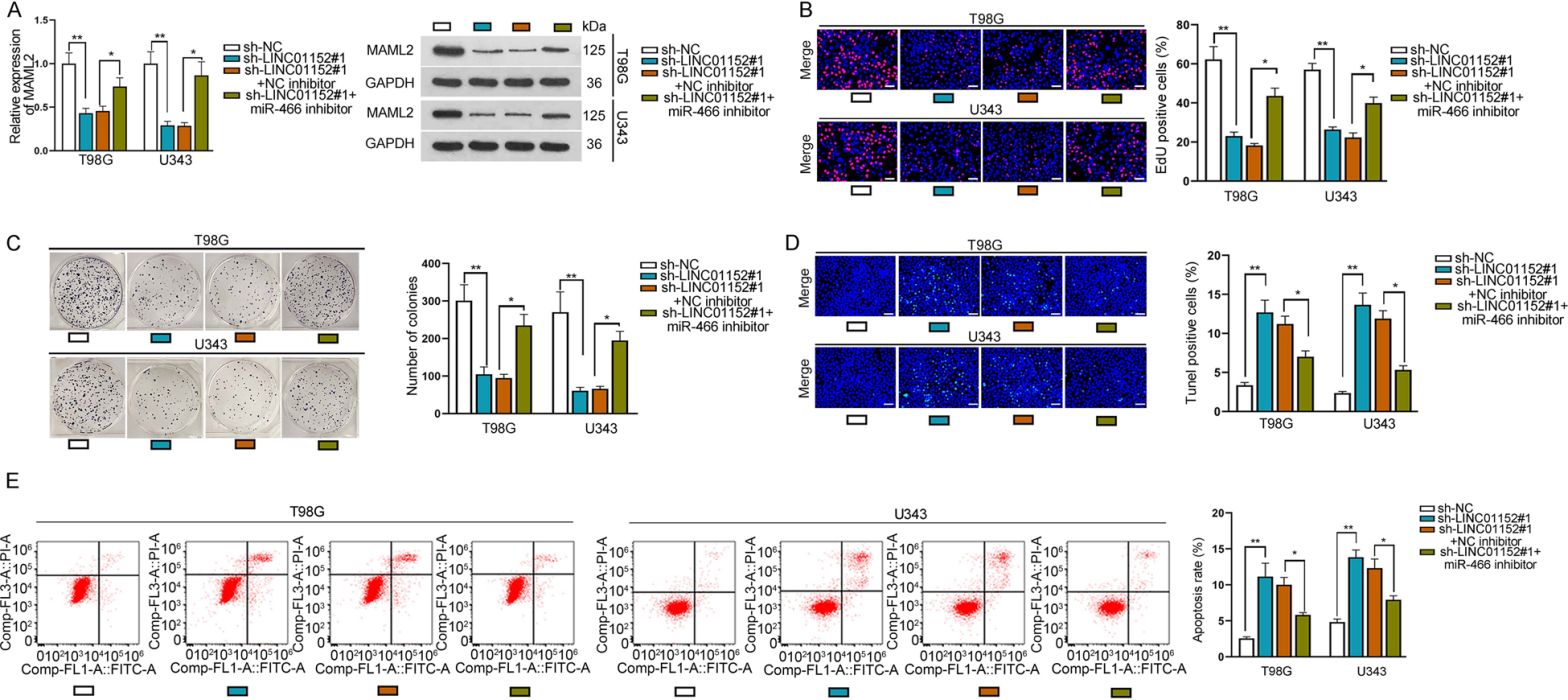
LINC01152/miR-466/MAML2 axis could modulate the progression of GBM cells. **A** Expression of MAML2 was determined via qRT-PCR and western blot in T98G and U343 cells with LINC01152 depletion or together with miR-466 inhibition. **B**, **C** EdU (scale bar = 100 μm) assay and colony formation assay assessed the proliferation of T98G and U343 cells under indicated conditions. **D**, **E** TUNEL (scale bar = 120 μm) assay and flow cytometry analysis investigated the apoptosis of indicated T98G and U343 cells. *P < 0.05, **P < 0.01.

Furthermore, we identified the function of LINC01152/miR-466 axis in GBM cells. It was validated that the mRNA expression and protein level of MAML2 in T98G and U343 cells were both inhibited by silenced LINC01152 and then partially regained via knockdown of miR-466 (Fig. 4A). Also, cell proliferation was reduced by LINC01152 depletion and partially offset under miR-466 inhibition, according to the results of EdU assay and colony formation assay (Fig. 4B, C). Meanwhile, cell apoptosis elevated by silenced LINC01152 was partially countervailed after inhibiting miR-466, as evidenced by the outcomes of TUNEL assay and flow cytometry analysis (Fig. 4D, E). In a word, LINC01152 partially relied on miR-466 to modulate GBM cellular processes.

### LINC01152 also contributed to MAML2 elevation in GBM via recruiting SRSF1

**Fig. 5:**
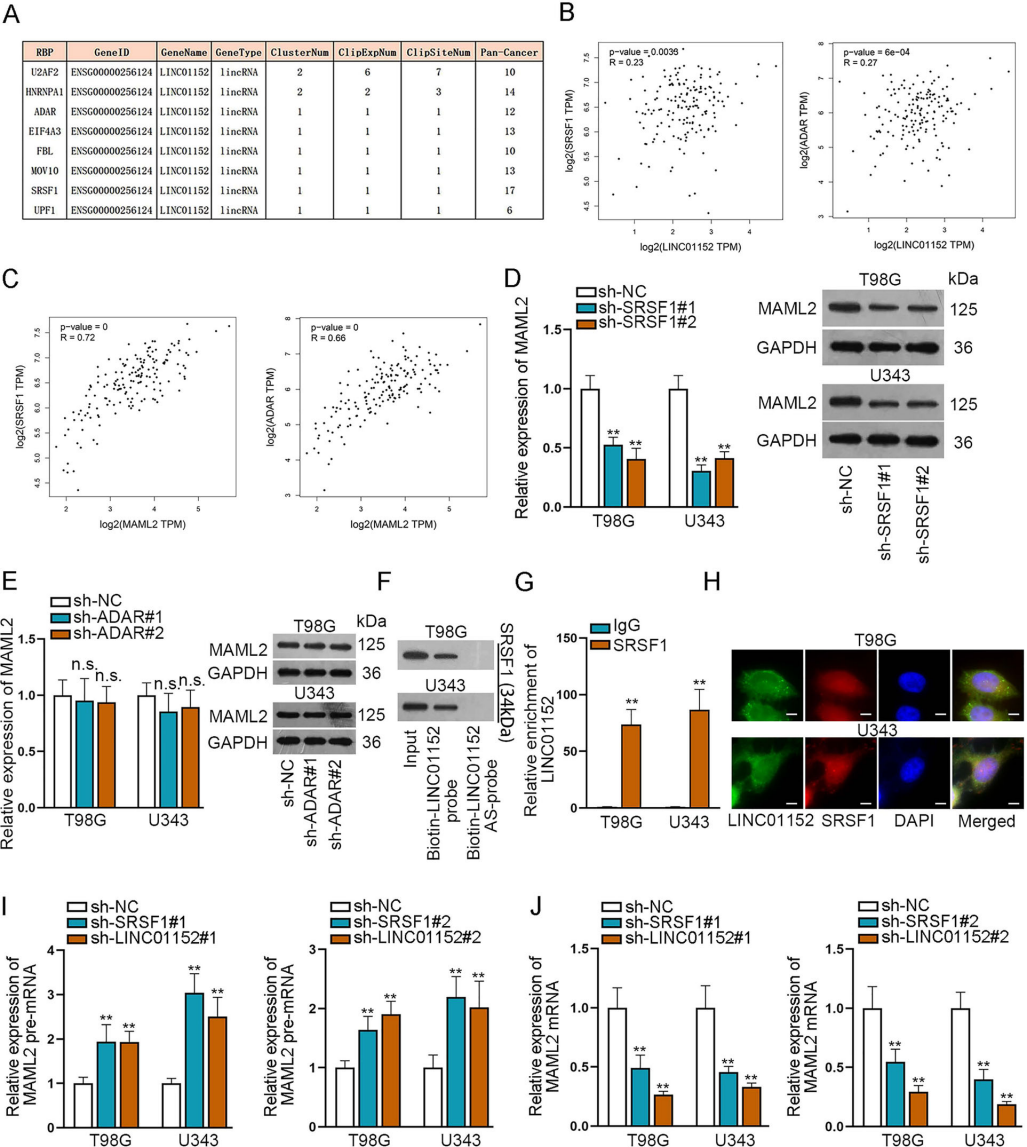
LINC01152 interacted with SRSF1 to boost MAML2 expression in GBM cells. **A** Potential RNA binding proteins were searched via ENCORI database. **B**, **C** Correlation analysis in GEPIA database was implemented to search the correlation of LINC01152 and MAML2 with SRSF1 and ADAR. **D**, **E** Expression of MAML2 was assessed via qRT-PCR and western blot in T98G and U343 cells when SRSF1 or ADAR was inhibited. **F**, **G** RNA-protein pull down assay and RIP assay searched the binding between LINC01152 and SRSF1. **H** FISH assay (scale bar = 15 μm) identified the co-location of LINC01152 and SRSF1 in T98G and U343 cells. **I**, **J** Expression of MAML2 pre-mRNA and MAML2 mRNA was detected via qRT-PCR in T98G and U343 cells when SRSF1 or LINC01152 was silenced. **P < 0.01, n.s.: no significance.

Considering the partial rescue results of LINC01152/miR-466 axis, we predicted there were RNA binding proteins involving in the LINC01152/miR-466 axis modulation. Eight potential RNA binding proteins were searched via ENCORI database (Fig. 5A). Correlation analysis in GEPIA database was implemented to search the relation of LINC01152 and MAML2 to these eight proteins. Results found that the expression correlation of LINC01152 and MAML2 with serine and arginine-rich splicing factor 1 (SRSF1) and adenosine deaminase RNA specific (ADAR) were most significant (Fig. 5B, C and Fig. S2A, B). Then, we silenced SRSF1 and ADAR using specific shRNAs and detected the inhibition efficiencies via qRT-PCR and western blot (Fig. S2C, D). As a result, the expression of MAML2 was identified to be affected only by SRSF1 rather than ADAR (Fig. 5D, E). Moreover, the results of RNA-protein pull down assay and RIP assay certified the interaction between SRSF1 and LINC01152 in T98G and U343 cells (Fig. 5F, G). Meanwhile, FISH assay also indicated the co-localization of LINC01152 and SRSF1 mainly in cell nucleus (Fig. 5H). Importantly, we discovered that the level of MAML2 pre-mRNA was elevated and that of mature MAML2 mRNA was reduced in T98G and U343 cells when SRSF1 or LINC01152 was silenced (Fig. 5I, J), indicating the participation of SRSF1 in the shearing of MAML2 in the nucleus of GBM cells. In conclusion, LINC01152 recruited SRSF1 to facilitate MAML2 expression in GBM cells.

### MAML2 could activate the LINC01152 expression via modulating Notch pathway in GBM cells

**Fig. 6:**
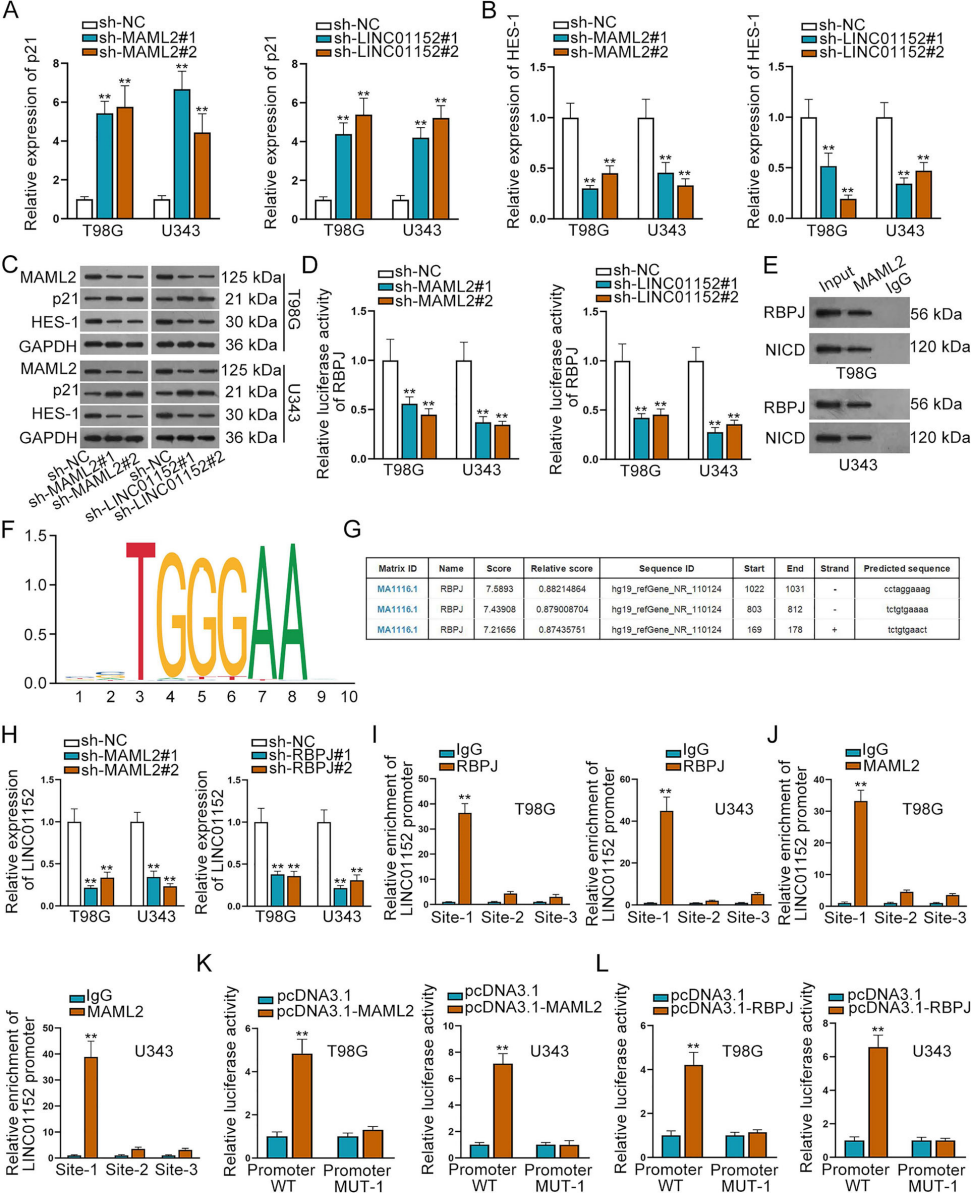
MAML2 could activate LINC01152 expression via modulating Notch pathway in GBM cells. **A**–**C** Expression of p21 and HES-1 was tested via qRT-PCR and western blot when MAML2 or LINC01152 was silenced in T98G and U343 cells. **D** Luciferase activity of RBPJ was detected via luciferase reporter assay in T98G and U343 cells when MAML2 or LINC01152 was silenced. **E** CoIP assay searched the binding of MAML2 to RBPJ and NICD. **F**, **G** JASPAR (http://jaspar.genereg.net/) database was used to search the DNA motif of RBPJ and possible binding sites between RBPJ and LINC01152 promoter. **H** qRT-PCR probed the expression of LINC01152 when MAML2 and RBPJ were silenced. **I**, **J** The binding of RBPJ and MAML2 to LINC01152 promoter was verified via ChIP assay in T98G and U343 cells. **K**, **L** Luciferase activity of LINC01152 promoter (wild-type or mutant) was tested via luciferase reporter assay when RBPJ or MAML2 was overexpressed. **P < 0.01.

MAML family is one of the regulatory elements of Notch pathway activation^19^. For searching whether LINC01152/MAML2 axis modulated Notch pathway in GBM, we detected their impact on the downstream target genes of Notch pathway. Interestingly, we discovered that both mRNA and protein levels of p21 were enhanced whereas the expression of HES-1 were decreased by silenced LINC01152 or MAML2 (Fig. 6A–C). Meanwhile, we also found that the luciferase activity of RBPJ, the downstream transcription factor of Notch pathway, was significantly reduced by LINC01152 or MAML2 inhibition (Fig. 6D), which proved that LICN01152/MAML2 axis could activate the pathway of Notch.

Since Notch pathway could affect the expression of diverse genes, we then wondered whether there was a feedback regulation between LINC01152 and this pathway. Intriguingly, we proved that inhibition of NOTCH1 led to declined level of LINC01152 in both T98G and U343 cells (Fig. S3A, B). Additionally, the level of active NOTCH1 protein val1744 was enhanced in GBM cells but not in LGG cells (Fig. S3C), and the alteration trends were similar to that of LINC01152 expression in these cells. Moreover, suppressing NOTCH1 could normalize the impacts of upregulated LINC01152 on LINC01152 expression and GBM cell functions (Fig. S3D–G). Thus, we speculated that Notch pathway might regulate LINC01152 transcription in turn. Subsequently, CoIP assay results unveiled that MAML2 could bind to RBPJ and NICD to form a transcription complex (Fig. 6E). Then, DNA motif of RBPJ and possible binding site of RBPJ to LINC01152 promoter were presented according to JASPAR (http://jaspar.genereg.net/) database (Fig. 6F, G). Inhibition efficiency of RBPJ was tested via qRT-PCR and western blot in T98G and U343 cells (Fig. S4A). As a consequence, qRT-PCR probed the expression of LINC01152 was evidently lessened when MAML2 or RBPJ was silenced (Fig. 6H). Furthermore, the results of ChIP assay indicated that MAML2 and RBPJ could bind to LINC01152 promoter in the predicted site 1 (Fig. 6I, J). Thereafter, we overexpressed MAML2 and RBPJ in T98G and U343 cells, as tested by qRT-PCR and western blot (Fig. S4B, C). In the outcomes of luciferase reporter assay, we found the luciferase activity of wild type LINC01152 promoter not the mutant promoter was obviously increased by overexpressed RBPJ and MAML2 (Fig. 6K, L), which indicated that RBPJ and MAML2 could activate the transcription of LINC01152. In a word, MAML2 transcriptionally activate the LINC01152 expression via modulating Notch pathway in GBM cells.

### LINC01152/MAML2 axis could upregulate the progression of GBM

**Fig. 7:**
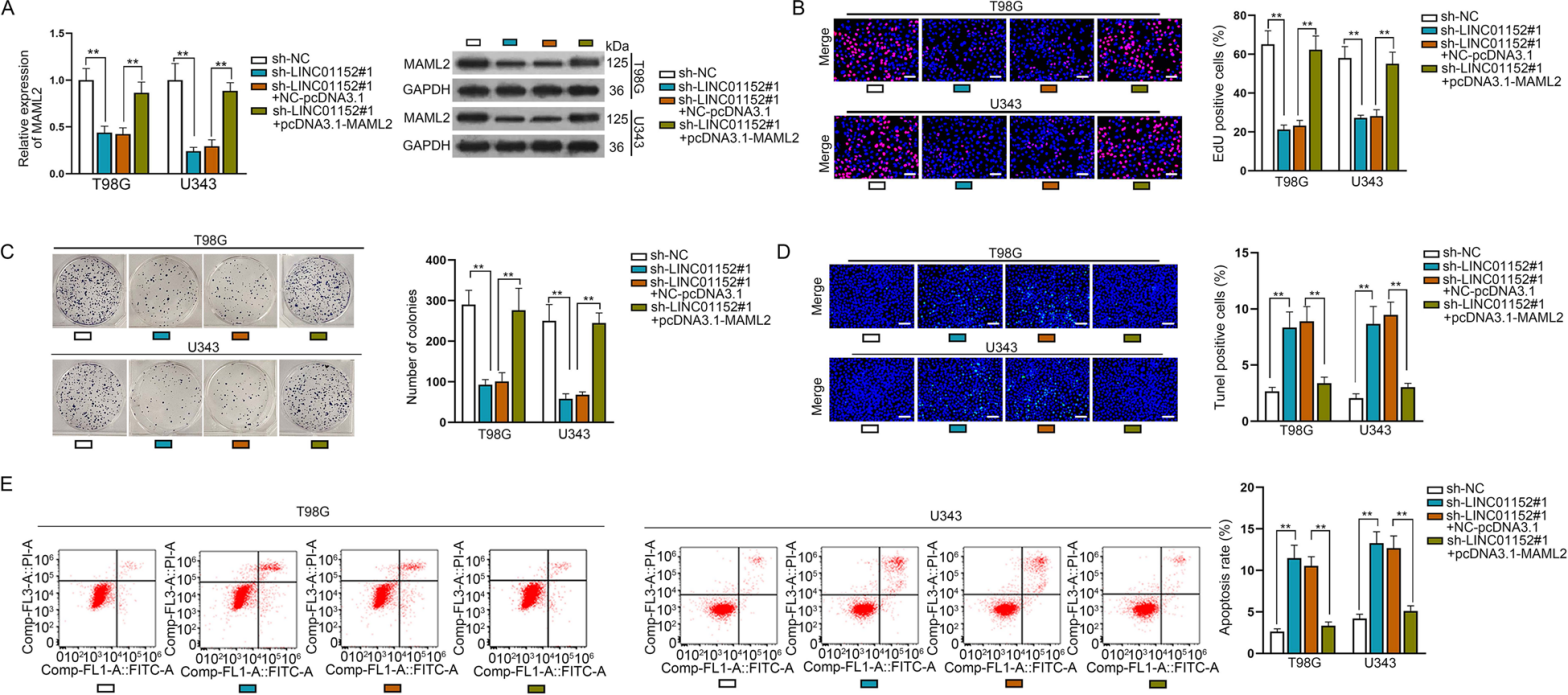
LINC01152/MAML2 axis could facilitate the progression of GBM. **A** qRT-PCR and western blot searched the mRNA and protein levels of MAML2 in LINC01152-silenced T98G and U343 cells with or without MAML2 overexpression. **B**, **C** EdU (scale bar = 100 μm) assay and colony formation assay assessed the proliferation of T98G and U343 cells in different groups. **D**, **E** TUNEL (scale bar = 120 μm) assay and flow cytometry analysis investigated the apoptosis of indicated T98G and U343 cells. **P < 0.01.

For the next step, we searched the effect of LINC01152/MAML2 signaling on the function of GBM cells through rescue assays. It was proved that MAML2 expression at both mRNA and protein levels was reduced by silenced LINC01152 and fully regained after overexpressing MAML2 (Fig. 7A). In addition, the inhibition on cell proliferation by silenced LINC01152 was fully countervailed by overexpressed MAML2 (Fig. 7B, C). Moreover, the outcomes of TUNEL assay and flow cytometry analysis demonstrated that cell apoptosis was facilitated by inhibited LINC01152 and fully normalized under MAML2 enhancement (Fig. 7D, E). Furthermore, in vivo rescue experiments were also implemented. It manifested that the restrained tumor growth owing to LINC01152 depletion was partly reversed by inhibited miR-466 but completely recovered by upregulated MAML2 (Fig. S4D–F). Such phenomena resulted from partial restoration of MAML2 expression in response to miR-466 inhibition and complete recovery of MAML2 expression in face of MAML2 upregulation (Fig. S4G). Thence, we concluded that LINC01152 contributed to the progression of GBM by targeting MAML2.

## Discussion

GBM has caused significant physical and psychological pain in GBM patients, because of the poor prognosis and low overall survival rate^20^. With the discovery of lncRNA function in human cancer, much more effective target therapies for cancer have been identified^21^. However, the treatment for GBM is still not optimistic. Previous studies have proved the participation of lncRNAs in GBM progression. For instance, LINC00152 can activate cell growth and invasion, and EMT via reducing the expression of miR-107 in GBM^22^. Also, SNHG20 can enhance cell proliferation and stemness in GBM by modulating PI3K/Akt/mTOR pathway^23^. Besides, LINC01446 can facilitate GBM progression via sponging miR-489-3p^24^. In our study, we scrutinized the role of LINC01152 in GBM. In the beginning, LINC01152 expression was identified to be upregulated in GBM tissues and cells. Meanwhile, previous study has studied the oncogenic role of LINC01152 in hepatocellular carcinoma^14^. Therefore, we scrutinized the function of LINC01152 in GBM. Consistently, we discovered that cell proliferation was hampered while cell apoptosis was stimulated in response to silenced LINC01152. Meanwhile, the outcomes of in vivo experiments indicated that GBM tumor formation ability was obviously inhibited by LINC01152 depletion. In a word, we identified LINC01152 as an oncogene in GBM.

Then, among top three protein-coding genes associated with LINC01152 in GBM via GEPIA database, MAML2 expression was also identified to present high level in GBM tissues. Previously, MAML2 has been reported to be upregulated and function as an tumor-activator in mucoepidermoid carcinoma and breast cancer^17,18^. In our work, functional assays were conducted to verify the function of MAML2 in GBM, and we found that MAML2 could play as an oncogene in GBM as well.

Furthermore, we found that LINC01152 depletion could reduce the expression of MAML2 in GBM cells at post-transcriptional level. Besides, LINC01152 mainly distributed in the cytoplasm of GBM cells. Thence, we predicted that LINC01152 might be involved in a ceRNA network to modulate the expression of MAML2 in GBM. LncRNAs have been reported to function as a ceRNA to affect the expression of protein-coding genes through sponging microRNAs (miRNAs). For example, lncRNA FAL1 can activate the progression of hepatocellular carcinoma by competitively binding to miR-1236^25^. TTN-AS1 can facilitate the metastasis of lung adenocarcinoma by regulating miR-142-5p and CDK5^26^. Also, LINC00483 can modulate cell growth and apoptosis in gastric cancer by sponging miR-30a-3p to upregulate SPAG9 and activate MAPK pathway^27^. In our study, miR-466 was found to bind to both LINC01152 and MAML2, and it was lowly expressed in GBM cells. However, inhibiting miR-466 could only partly rescue the effect of depleted LINC01152 on GBM cellular functions, indicating the ceRNA network was not the only mechanism involved in the modulation of LINC01152 on MAML2. Thence we speculated whether another common role of lncRNAs, recruiting RBPs, was also applicable for LINC01152 in regulating MAML2 in GBM^28^. For example, HULC can weaken the chemosensitivity of hepatocellular carcinoma by stabilizing Sirt1^29^. In our study, we searched out SRSF1 as the RBP that can bind to LINC01152, so as to and activate the expression of MAML2 in GBM.

Furthermore, we detected that Notch signaling pathway was affected by MAML2. Notch signaling pathway has been identified to be activated to facilitate the progression of multiple cancers^30^. For example, Syndecan-1 can activate triple negative inflammatory breast cancer via regulating IL-6/STAT3, Notch and EGFR signaling pathways^31^. In our study, we found that inhibiting MAML2 and LINC01152 could inactivate Notch signaling pathway significantly. In turn, suppressing NOTCH1 could also lead to silenced LINC01152, so as to reverse the facilitating function of overexpressed LINC01152 in GBM. Intriguingly, the trend of active NOTCH1 level in GBM cell lines was similar to that of LINC01152 expression. Moreover, we proved that NICD/RBPJ/MAML2 transcriptional complex could activate the transcription of LINC01152 in GBM cells. In the end, the results of in vivo assays evidenced that LINC01152 depended on MAML2 to contribute to GBM tumorigenesis, while partially relied on miR-466.

**Fig. 8:**
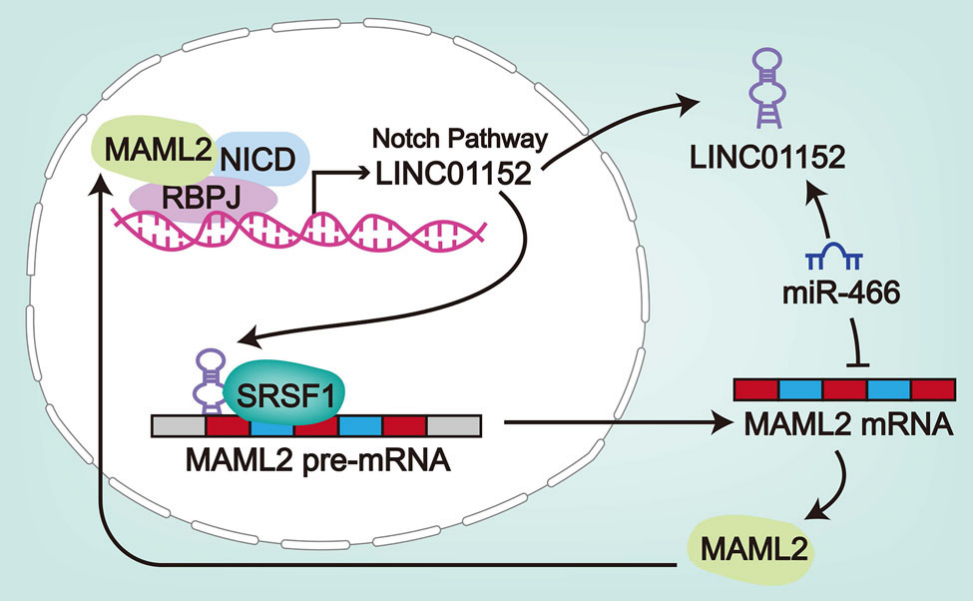
A positive feedback loop between LINC01152 and NOTCH pathway promotes GBM cell growth. The schematic diagram describing the molecular mechanism of LINC01152 in GBM cells.

Taken together, our study firstly scrutinized the molecular mechanism of LINC01152 in aggravating GBM development. LINC01152 could sponge miR-466 and recruit SRSF1 to activate MAML2, therefore, facilitating GBM progression via Notch signaling pathway (Fig. 8).

## Supplementary information

Supplementary Figure legends

Supplementary Figure 1

Supplementary Figure 2

Supplementary Figure 3

Supplementary Figure 4
