# Correction: LINC01152 upregulates MAML2 expression to modulate the progression of glioblastoma multiforme via Notch signaling pathway

**DOI:** 10.1038/s41419-021-04106-8

**Published:** 2021-09-23

**Authors:** Jianheng Wu, Nannan Wang, Ying Yang, Guangyuan Jiang, Qingchun Mu, Hui Zhan, Fuyong Li

**Affiliations:** 1 Department of Neurosurgery, Gaozhou People’s Hospital, Gaozhou, 525200 Guangdong China; 2 Department of Gastroenterology, Gaozhou People’s Hospital, Gaozhou, 525200 Guangdong China; 3 Electroencephalogram Room, Weifang Yidu Central Hospital, Weifang, 262500 Shandong China; 4 Department of Neurosurgery, Nanxishan Hospital of Guangxi Zhuang Autonomous Region, Guilin, 541000 Guangxi China; 5 Department of Neurosurgery, the People’s Hospital of China Medical University (the People’s Hospital of Liaoning Province), Shenyang, 110016 Liaoning China

**Keywords:** Cancer stem cells, Cell biology, Biomarkers

Correction to: *Cell Death and Disease* (2021) 12:115; 10.1038/s41419-020-03163-9, published online 22 January 2021

Since the publication of the article a request has been made to change the authorship of the article. The proposed new authorship is: Jianheng Wu, Nannan Wang, Ying Yang, Guangyuan Jiang, Hui Zhan, Fuyong Li. Qingchun Mu was removed as an author. The original article has been corrected.

