# Supplementary material for: LINC01152 upregulates MAML2 expression to modulate the progression of glioblastoma multiforme via Notch signaling pathway
Source: Cell Death Dis. 2021 Jan 22;12(1):115. doi: 10.1038/s41419-020-03163-9 (PMC7822850; doi:10.1038/s41419-020-03163-9)
Supplement: Supplementary file 1 — Supplementary Figure legends [file 41419_2020_3163_MOESM1_ESM.docx]

**Supplementary Figure 1**

A. Expression of LINC01152 in 518 LGG (brain lower grade glioma) samples and 207 normal tissues was identified via GEPIA database.

B. Expression of LINC01152 in normal human astrocytes cell lines (NHA) and LGG cell lines (LN-215 and U138) was detected via qRT-PCR.

C. Expression of STAC2 or BBS2 in 163 GBM tissues and 207 normal tissues was searched via GEPIA database.

D. The expression of MAML2 in 38 pairs of GBM samples was determined via qRT-PCR.

E. Luciferase activity of MAML2 promoter was assessed via luciferase reporter assay when LINC01152 was silenced in T98G and U343 cells.

F. The level of miR-466 in 38 pairs of GBM samples was tested via qRT-PCR.

G-H. Overexpression efficiency of miR-466 or LINC01152 was tested in T98G and U343 cells via qRT-PCR. ^**^P < 0.01, n.s.: no significance.

**Supplementary Figure 2**

A. The correlation of UPF1, EIF4A3 U2AF2, MOV10, FBL and HNRNPA1 with LINC01152 in GBM tissues was presented; data were all obtained from GEPIA database.

B. The relation of UPF1, EIF4A3 U2AF2, MOV10, FBL and HNRNPA1 to MAML2 was presented, and the data were all obtained from GEPIA database.

C-D. Inhibition efficiency of SRSF1 and ADAR was assessed via qRT-PCR and western blot in T98G and U343 cells. ^**^P < 0.01.

**Supplementary Figure 3**

A-B. qRT-PCR evaluated the expression of NOTCH1 and LINC01152 in T98G and U343 cells transfected with sh-NC or sh-NOTCH1#1/2.

C. The protein level of val1744 (cleaved NOTCH1) in GBM and LGG cell lines was examined by western blot.

D-E. LINC01152 expression in T98G and U343 cells transfected with pcDNA3.1/LINC01152 or together with sh-NOTCH1#1 was determined via qRT-PCR.

F-G. EdU and colony formation assays assessed the proliferation of GBM cells with above transfections. ^**^P < 0.01.

**Supplementary Figure 4**

A. Inhibition efficiency of RBPJ was tested via qRT-PCR and western blot in T98G and U343 cells.

B-C. qRT-PCR and western blot tested overexpression efficiency of MAML2 and RBPJ in T98G and U343 cells.

D. Representative images of in vivo tumors derived from cells transfected with sh-NC, sh-LINC01152#1, sh-LINC01152#1+antagomir-466, or sh-LINC01152#1+pcDNA3.1/MAML2.

E. The growth curve of tumors from above four groups.

F. Tumor weight under different contexts.

G. The level of MAML2 in these four kinds of in vivo tumors was estimated by qRT-PCR. ^*^P < 0.05, ^**^P < 0.01.
