# Supplementary figures and images for: LINC01152 upregulates MAML2 expression to modulate the progression of glioblastoma multiforme via Notch signaling pathway
Source: Cell Death Dis. 2021 Jan 22;12(1):115. doi: 10.1038/s41419-020-03163-9 (PMC7822850; doi:10.1038/s41419-020-03163-9)

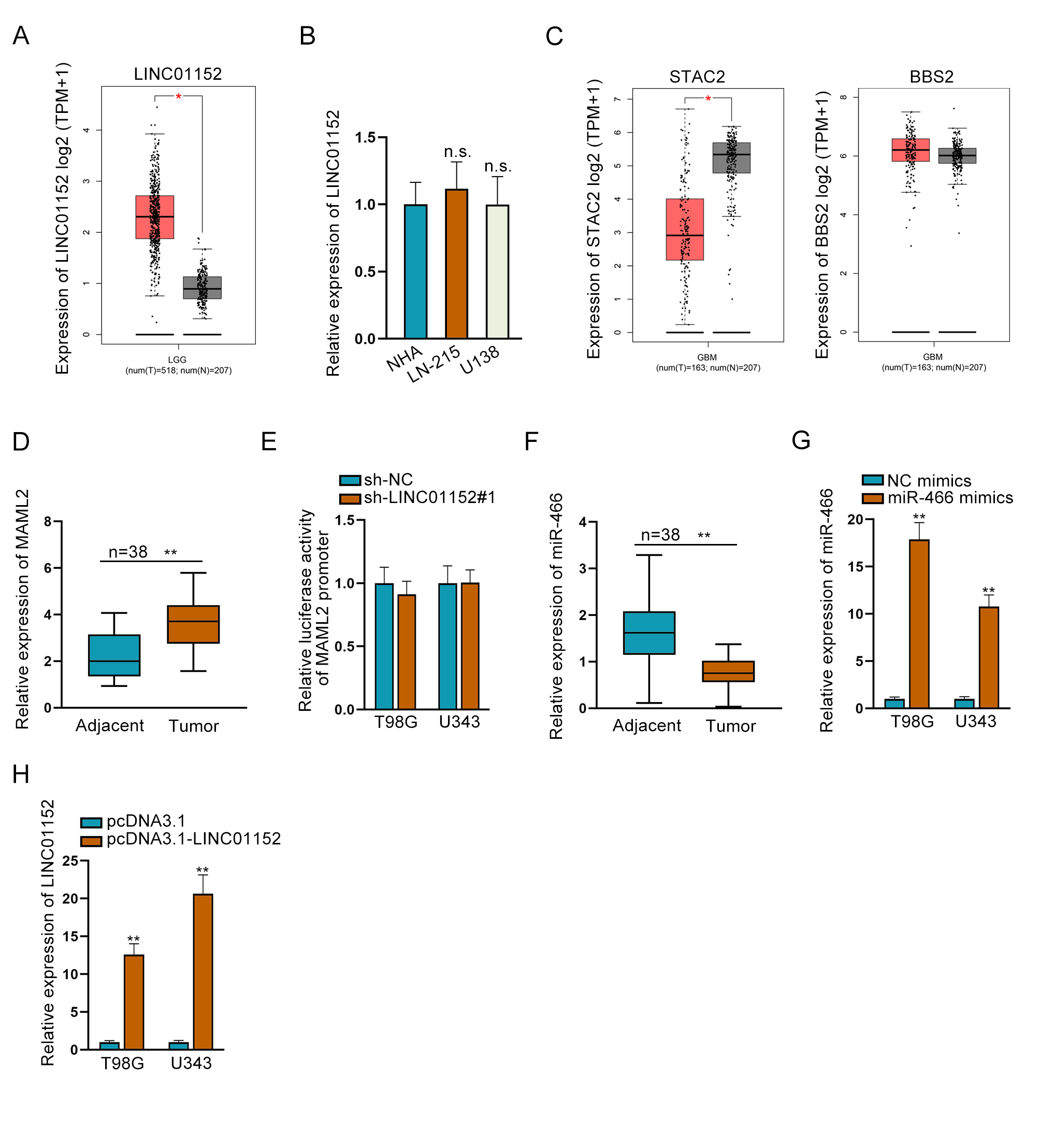

Supplement: Supplementary file 2 — Supplementary Figure 1 [file 41419_2020_3163_MOESM2_ESM.tif]

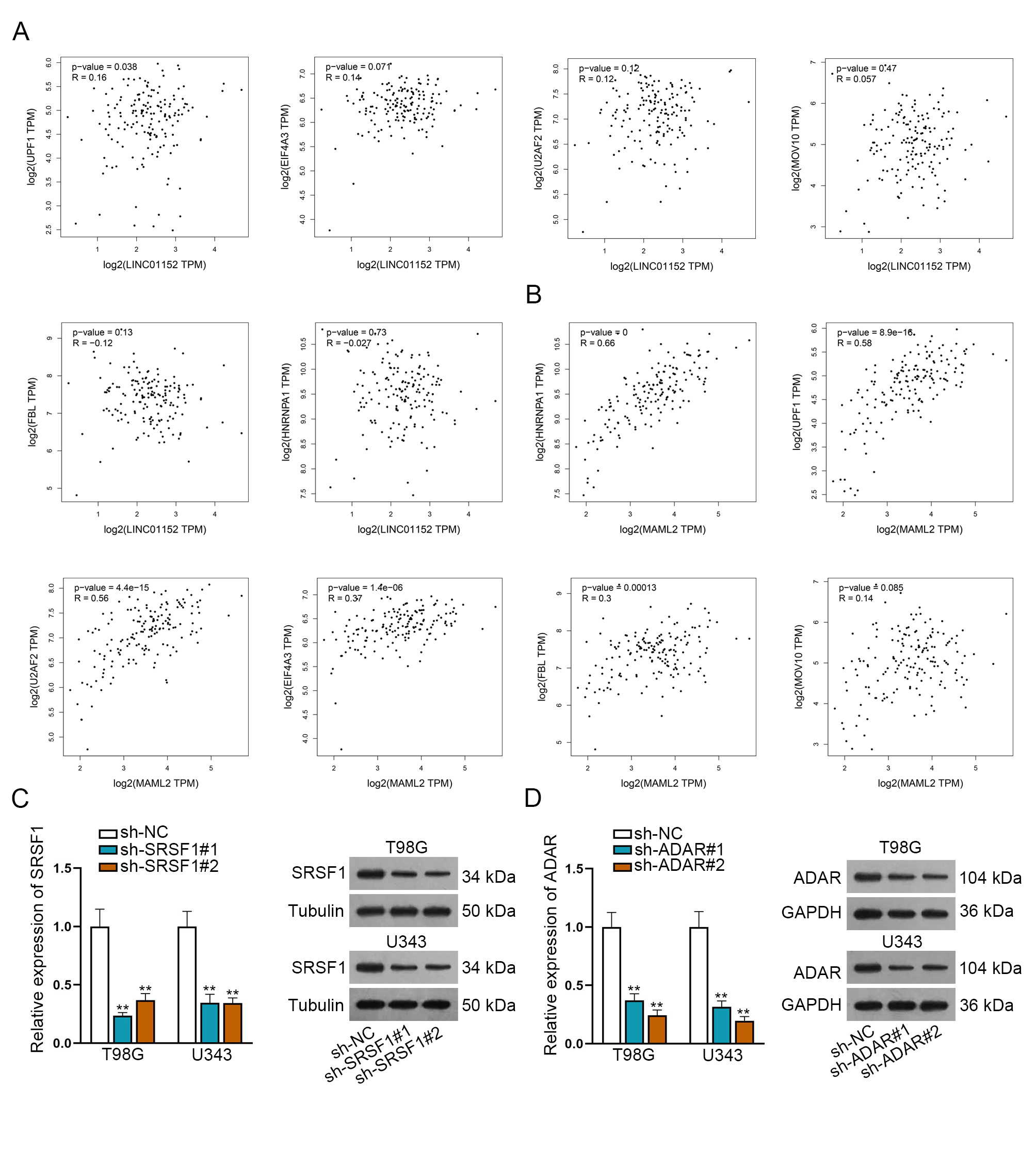

Supplement: Supplementary file 3 — Supplementary Figure 2 [file 41419_2020_3163_MOESM3_ESM.tif]

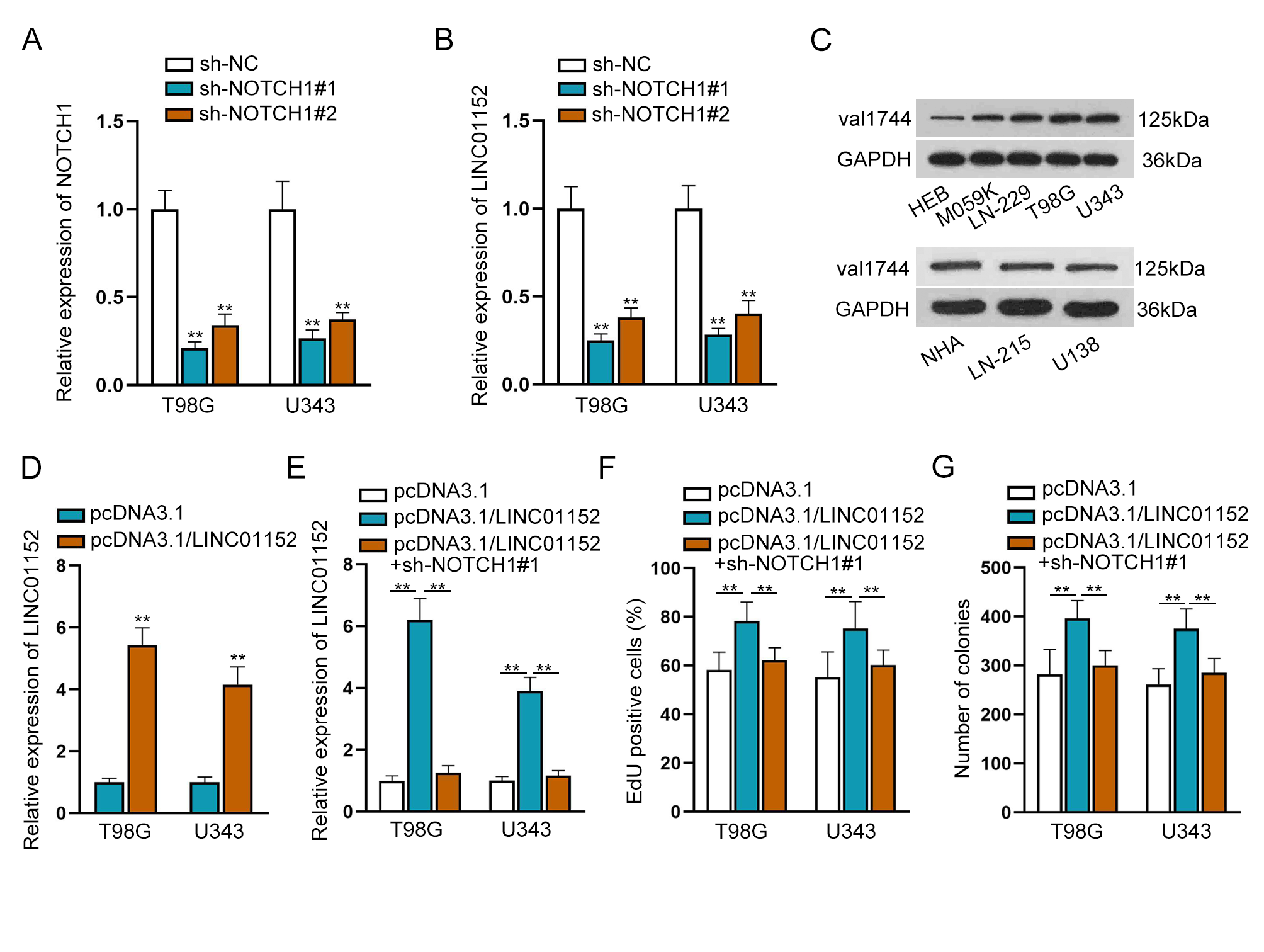

Supplement: Supplementary file 4 — Supplementary Figure 3 [file 41419_2020_3163_MOESM4_ESM.tif]

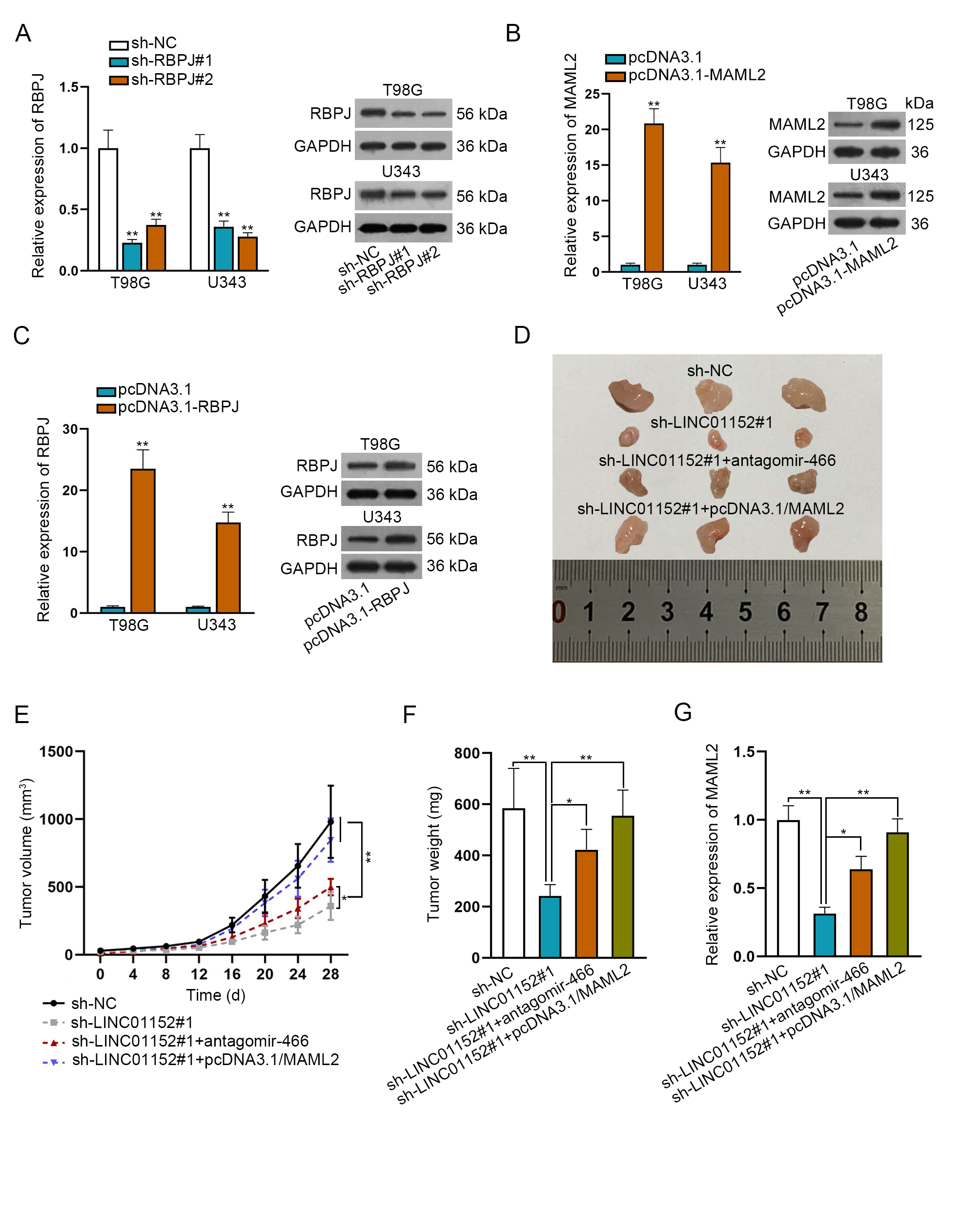

Supplement: Supplementary file 5 — Supplementary Figure 4 [file 41419_2020_3163_MOESM5_ESM.tif]
